# Antiviral Potential of *Spiraea* Extracts (Prepared by Repercolation) Against Influenza A (H1N1) Virus

**DOI:** 10.3390/foods13244008

**Published:** 2024-12-11

**Authors:** Vera A. Kostikova, Yana L. Esaulkova, Polina A. Ilyina, Vladimir V. Zarubaev, Vladimir V. Sheikin, Anastasia A. Petruk, Ekaterina D. Rubtsova, Tatiana N. Veklich

**Affiliations:** 1 Central Siberian Botanical Garden, Siberian Branch of Russian Academy of Sciences (CSBG SB RAS), Novosibirsk 630090, Russia; pet.a@mail.ru (A.A.P.); katiarub7558@gmail.com (E.D.R.); 2 St. Petersburg Pasteur Institute, St. Petersburg 197101, Russia; ianaesaulkova@gmail.com (Y.L.E.); il401polina@gmail.com (P.A.I.); zarubaev@gmail.com (V.V.Z.); 3 Department of Pharmaceutical Technology and Biotechnology, Siberian State Medical University, Tomsk 634050, Russia; sheykinvv@gmail.com; 4 Amur Branch of Botanical Garden-Institute, Far Eastern Branch of Russian Academy of Sciences, Blagoveshchensk 675000, Russia; tbliznjuk@mail.ru; 5 Zeya State Nature Reserve, Zeya 676246, Russia

**Keywords:** *Spiraea*, repercolation, antiviral activity, biologically active compound

## Abstract

An antiviral effect of extracts prepared from aerial parts of nine species and from leaves of two species of the genus *Spiraea* L. was investigated for potential antiviral activity toward influenza A (H1N1) virus. The toxicity of dry extracts was analyzed, and the most selective extract was identified in vitro. The study’s material was collected in the Asian part of Russia. The plant extracts were prepared via three-stage countercurrent repercolation involving a complete cycle. All 40%-ethanolic extracts from *Spiraea* manifested antiviral activity against influenza A (H1N1) virus, with a selectivity index (SI) ranging from 1 to 10. IC_50_ values indicated that the *S. salicifolia* L. S15 leaf extract (5.9 µg/mL) has the most pronounced antiviral effect and the lowest toxicity (CC_50_ = 57.6 µg/mL) among the studied samples. The SI of this extract was 10, which exceeded that of the antiviral agent rimantadine (SI = 6). Biologically active compounds in the extract with the highest antiviral activity were identified using UV spectrometry and high-performance liquid chromatography. The *S. salicifolia* leaf extract was found to contain phenolic acids (chlorogenic, gentisic, caffeic, ferulic, and cinnamic acids), flavonols (quercetin, quercetin-3-glucuronoside, hyperoside, isoquercitrin, rutin, spiraeoside, avicularin, quercitrin, kaempferol, nicotiflorin, astragalin, and isorhamnetin-3-rutinoside), flavones (orientin, luteolin-7-glucoside, and vitexin), and coumarin. Predominant biologically active compounds in the *S. salicifolia* S15 leaf extract were such flavonols as rutin (19.3 mg/g), isoquercitrin (16.6 mg/g), isorhamnetin-3-rutinoside (10.6 mg/g), and astragalin (9.5 mg/g). Extraction of *S. salicifolia* leaves by repercolation is a more suitable method for extracting active ingredients with an antiviral effect.

## 1. Introduction

The selection of an appropriate extraction method (sample preparation method) is of paramount importance in any study on medicinal plants because this choice affects the study’s potential outcomes. Relevant biologically active phytochemicals are mainly bound to other compounds in plants. Therefore, several crucial factors associated with the extraction method, including the choice of plant material, solvent, pressure, temperature, and procedure duration, can affect the method’s performance. The advent of modern chromatographic and spectrometric techniques has made the procedure of extracting biologically active compounds relatively easy. Nonetheless, traditional plant extraction methods, such as maceration, percolation, and Soxhlet extraction, are employed in small-scale production or for research purposes [1]. The selection of the optimal method for extraction of biologically active compounds from plants is an important step in the search for potential sources of raw materials.

*Spiraea* L. (Rosaceae Juss.) is a genus of deciduous shrubs that come in a wide variety of shapes and sizes, with heights ranging from 0.15 to 2.50 m and erect spreading branches [2]. The genus *Spiraea* comprises light-loving shrubs that flourish in open spaces, stony mountain slopes, screes, river banks, and shrub steppes, where they often become dominant or codominant species [2,3]. Species of the genus *Spiraea* are used extensively worldwide as ornamental shrubs for urban and rural landscaping, as fodder, or as melliferous or soil-strengthening plants [4]. In addition, *Spiraea* plants are of interest due to their use in folk medicine and to high resource potential. A number of *Spiraea* species are employed in folk medicine of various Asian countries as antimalarial and anti-inflammatory agents [5,6,7]. In traditional Chinese medicine, fruits, leaves, and roots of *S. japonica* L. fil. and its lines are utilized as analgesic and diuretic agents [8,9]. Decoctions and infusions of *S. salicifolia* L. are used to treat gastrointestinal diseases, rheumatism, helminthiasis, gynecological diseases, and diabetes mellitus [7,10]. Extracts from *Spiraea* plants have been shown to have antioxidant [11], antitumor [12], and anti-inflammatory [13,14] properties and other beneficial biological effects.

As indicated in the literature, secondary metabolites with notable biological activity have been identified in plants of the genus *Spiraea*. These include flavonoids, phenolcarboxylic acids, tannins, coumarins, terpenoids, steroidal glycosides, cyanogenic glycosides, neolignans, fatty acids, and essential oils [15,16,17,18,19]. Some species of the genus *Spiraea* native to Southeast Asia accumulate diterpene alkaloids of atisine and hetisine types [20]. Among *Spiraea* plants growing in Russia, chemical composition and practical potential of widespread species—*S. salicifolia* and *S. media* F. Schmidt—have been the subject of the most comprehensive studies. There is little research on levels of secondary metabolites in many *Spiraea* species native to Russia. The biological activity of *Spiraea* species, such as *S. humilis* Pojark, *S. elegans* Pojark, *S. sericea* Turcz, *S. trilobata* L., and *S. ouensanensis* H. Lév., has been the subject of only limited research, which is likely due to their limited occurrence. Additionally, an analysis of previous studies has revealed that extracts prepared by hot maceration are commonly used to investigate the phytochemistry of *Spiraea* plants [21]. There is another method of extract preparation that is seldom employed. In particular, the antiviral activity of *Spiraea* plants has not been studied in extracts prepared by complete-cycle repercolation. Repercolation is the most widely used in pharmaceutical factories owing to its feasibility for small-scale production of liquid extracts. This method allows for maximal depletion of raw materials to obtain highly concentrated extracts [1].

Influenza is a human respiratory disease caused by the influenza virus. Virions of the influenza virus are enveloped and contain a segmented single-stranded negative-sense genome. Due to the low fidelity of the viral polymerase, virus progeny is highly variable in genome composition. This results in the rapid escape of the virus from an immune response and the selection of viral variants resistant to antiviral agents. Therefore, the search for (and development of) novel virus-inhibitory drugs with new targets and mechanisms of action is a high-priority goal in terms of human health. Numerous plant-source secondary metabolites, particularly those from the genus *Spiraea*, have been shown to possess virus-inhibitory properties. Our study aimed to conduct a comparative analysis of the phytochemical composition of extracts prepared via the repercolation from aerial parts and/or leaves of nine *Spiraea* taxa native to Russia and to evaluate their antiviral potential against the influenza virus by in vitro experiments.

This study aimed to conduct a comparative analysis of the phytochemical composition of extracts prepared via repercolation from aerial parts and (or) leaves from nine *Spiraea* taxa native to Russia.

## 2. Materials and Methods

### 2.1. Plant Material

Samples of *Spiraea* plants were collected in natural populations of Siberia and the Far East as well as after introduction into an experimental field of the Laboratory of Phytochemistry, the Central Siberian Botanical Garden (CSBG SB RAS; Novosibirsk, Russia; Table 1). Dried herbal samples were used for further analysis. Voucher specimens were deposited in the Plant Material Storage Room in the Laboratory of Phytochemistry, the CSBG SB RAS (Novosibirsk, Russia). Most extracts were prepared from flowers, leaves, and stems in a 1:1:1 ratio (Table 1). For some extracts, leaves and stems were used in a slightly larger amount. Whole branches, not disassembled into organs, were used to prepare *S. hypericifolia* L. extracts owing to the small size of flowers and leaves. Leaves alone were used to prepare *S. media* and *S. salicifolia* extracts. Air-dry plant material was crushed in an A11 basic mill (IKA, Staufen im Breisgau, Germany) and sifted through a 5 mm sieve.

### 2.2. Extract Preparation 

A liquid extract was obtained in glass diffusers by three-stage countercurrent complete-cycle repercolation in a 1:1 ratio of raw materials to the finished product [22]. A 40% ethyl alcohol solution served as an extractant. The resulting liquid extract was dried in a dry evaporation vacuum system (Labconco, RapidVap, Kansas City, MO, USA) at 35 °C under pressure not exceeding 50 mBar. The residual moisture content of the obtained extracts was 2.9–3.8%.

To study biologically active compounds contained in the extracts from the aerial parts of *Spiraea* species, we dissolved 0.1 g of the thick extract in 10 mL of 40% aqueous ethanol.

### 2.3. Cells and Viruses

The Madin–Darby canine kidney cell line (MDCK, ATCC CCL-34) was used in this study. Cells were seeded in wells of 96-well plates (10^4^ cells per well in 0.1 mL of a culture medium) and ncubated until a confluent monolayer formed. Influenza virus strain A/Puerto Rico/8/34 (H1N1) from the collection of viral strains at St. Petersburg Pasteur Institute were propagated in the allantoic cavity of 9–11-day-old chicken embryos followed by determination of their activity by 50% tissue culture infectious dose (TCID_50_) titration on MDCK cells.

### 2.4. A Cytotoxicity Assay

The microtetrazolium (MTT) assay was employed to measure the cytotoxicity of the extracts. Briefly, three-fold serial dilutions of each extract (300 to 3.7 µg/mL) in MEM medium (Biolot, St. Petersburg, Russia) were prepared. MDCK cells (ATCC CCL-34) were incubated for 72 h at 36 °C and 5% CO_2_ in the presence of the diluted extracts. The degree of cell monolayer destruction was then evaluated using the MTT assay. The cells were washed twice with saline, and a solution of 3-(4,5-dimethylthiazolyl-2)-2,5-diphenyl tetrazolium bromide (ICN Biochemicals Inc., Aurora, OH, USA) (0.5 mg/mL) in phosphate-buffered saline was added into the wells. After one hour of incubation, the wells were washed, and the formazan residue was dissolved in dimethyl sulfoxide (0.1 mL per well). The optical density of the wells was measured on a Multiscan FC photometer (Thermo Scientific, Singapore) at a wavelength of 540 nm and plotted against the concentration of each extract. Each concentration was measured in three technical replicates. Next, the 50% cytotoxic concentration (CC_50_) of each extract (i.e., the extract dose that causes the death of 50% of the cells in culture or decreases optical density twofold as compared to control wells) was calculated from the obtained data.

### 2.5. A Cell Protection Assay

A monolayer of MDCK cells (10^4^/well) was washed with saline, and the extracts at appropriate concentrations in MEM (0.1 mL per well) were added to the wells. After one hour of incubation at 36 °C and 5% CO_2_, the cells were infected with the influenza virus A/Puerto Rico/8/34 (H1N1) (multiplicity of infection: 0.01 TCID_50_ per cell) and incubated for 72 h at 36 °C and 5% CO_2_. After that, cell viability was assessed using the MTT assay, as described above. The cytoprotective activity of the extracts was defined as their ability to increase optical density in comparison with control wells (containing a virus only, no drugs). Three independent experiments were performed on each extract. Based on the results, half-maximal inhibitory concentration (IC_50_), i.e., the concentration that results in 50% cell protection, was calculated for each extract. Additionally, the selectivity index (SI, the ratio of CC_50_ to IC_50_) was calculated for each extract. Clinically approved antivirals rimantadine (an inhibitor of viral proton pump M2) and oseltamivir (an inhibitor of viral neuraminidase: the enzyme essential for the budding of progeny virions) were used as reference compounds.

### 2.6. Quantification of Biologically Active Compounds in Spiraea Extracts

The total level of phenolic compounds was determined using the Folin–Ciocalteu reagent [23]. Absorption was measured at a wavelength of 765 nm on an SF-56 spectrophotometer (Lomo, Saint Petersburg, Russia). The standard curve was constructed with gallic acid (0.002–0.010 mg/mL), and the total phenolic content was expressed as mg of gallic acid equivalent per g of dry extract by means of the standard curve.

Flavonol concentration was determined by a spectrophotometric method based on the reaction of complex formation between flavonols and aluminum chloride [24]. The optical density of the solution with aluminum chloride was measured on the SF-56 spectrophotometer (Lomo, Saint Petersburg, Russia) at 415 nm in a cuvette with a light path of 1 cm, using an acetic acid solution as a control. The amount of flavonols in each sample was determined using a calibration curve built based on rutin (Chemapol, Mumbai, MH, India) at 0.002–0.010 mg/mL. The results were expressed as mg of rutin equivalent per g of dry extract with the help of the standard curve.

The concentration of catechins was determined spectrophotometrically by the method based on the ability of catechins to produce crimson coloration with a solution of vanillin in concentrated hydrochloric acid [25]. The intensity of colors was measured on SF-56 (Lomo, Saint Petersburg, Russia) at 504 nm in a cuvette with a light path of 1 cm. The standard curve was constructed with (±)-catechin (Sigma St. Louis, MO, USA) at 0.001–0.020 mg/mL. The catechin content was expressed as mg of (±)-catechin equivalent per g dry extract using the standard curve.

Quantitation of tannins (hydrolyzable tannins) was performed using the method proposed by L.M. Fedoseeva [26]. The intensity of the resulting color was measured using the SF-56 spectrophotometer (Lomo, Saint Petersburg, Russia) at 420 nm in a cuvette with a light path of 1 cm. A government standard sample of tannin (Sigma, St. Louis, MO, USA. concentration of 0.002–0.01 mg/mL) served as a standard. The results were expressed as mg of tannin equivalent per g of dry extract via the standard curve.

The total concentration of phenolic acids was determined with Arnov’s reagent [27,28]. The optical density was measured immediately at 490 nm on the SF-56 spectrophotometer (Lomo, Saint Petersburg, Russia). The results were expressed as mg of caffeic acid (Serva, Heidelberg, Germany, at 0.02–0.10 mg/mL) equivalents per g of dry extract using the standard curve.

A more detailed description of the methods is given in [12].

### 2.7. HPLC Assays of the Profile and Levels of Phenolic Compounds in Spiraea Extracts

The phenolic compounds in the eluate were analyzed by means of an analytical HPLC system that comprises an Agilent 1200 liquid chromatograph (Santa Clara, CA, USA) with a diode array detector, an autosampler, and a ChemStation system for collecting and processing chromatographic data.

The substances were separated via gradient elution on a Diasfer-110-C18 column, 4.6 × 150 mm in size, with a particle diameter of 5 μm. For the analysis of aqueous-ethanolic extracts, the system was utilized with the concentration of methanol in an aqueous solution of orthophosphoric acid (0.1%) in the mobile phase, changing from 19% to 70% during 30 min and then going up to 100% at 32 min. The eluent flow rate was 1 mL/min. The column temperature was 25 °C. The volume of the injected sample was 10 μL. Detection was performed at wavelengths λ = 255, 270, 290, 325, 340, 350, 360, and 370 nm. Methyl alcohol (special purity grade), orthophosphoric acid (special purity grade), and double-distilled water were used to prepare the mobile phases. Quantification of phenolic compounds was performed as reported previously [12]. Gentisic and cinnamic acids (Serva), chlorogenic and caffeic acids, quercetin, kaempferol, nicotiflorin, orientin, isorhamnetin-3-rutinoside, quercetin-3-glucuronoside, coumarin, luteolin-7-glucoside and quercitrin (Sigma-Aldrich, St. Louis, MO, USA), ferulic acid, isoquercitrin, avicularin, astragalin, rutin, spiraeoside, hyperoside, and vitexin (Fluka, Everett, WA, USA) were employed to prepare standard samples. The standard solutions had a concentration of 10 µg/mL.

## 3. Results and Discussion

### 3.1. Antiviral Activities in Extracts of Plants from the Genus Spiraea

The high potential of antiviral activity in plants of the family Rosaceae Juss has yet to be taken advantage of. Previously, a polyphenol-rich extract of *Rubus coreanus* Miq. Seeds—and gallic acid identified in them—have shown an ability to bind to viral hemagglutinin and completely inhibit the replication of influenza viruses regardless of type and subtype and have exerted a destructive effect on virions [29]. In that work, the strong protective effect of the extract was confirmed in vivo, mortality rates were reduced by 100%, and the infectious titer of the virus in lung tissue decreased by 3–4 orders of magnitude. These results were observed at very low doses (1–15 mg/kg) [29]. Another study has shown a similar activity of a Chinese quince extract [*Chaenomeles sinensis* (Dum. Cours.) Koehne] and of its anthocyanin fraction [30]. The antiviral activity of the *Spiraea* plants analyzed in our work is a relatively understudied area. Extracts of certain *Spiraea* species have been found to possess an antiviral effect against influenza viruses [31,32,33]. A comparative study on the antiviral and antioxidant potential has been conducted elsewhere, and the profile and levels of phenolic compounds were determined there in 70% water-ethanol extracts from aerial parts of three *Spiraea* species. Their results revealed moderate antiviral activity toward influenza viruses A and B (SI = 3–21) for the *S. media* and *S. hypericifolia* extracts, whereas the *S. salicifolia* extract manifested the highest activity against the DPPH radical (IC_50_ of 38.3 and 35.5 μg/mL) [34]. All previous studies on the antiviral potential of *Spiraea* plants have involved extracts prepared by hot maceration in a water bath. In our study, a new method of countercurrent three-stage repercolation involving a complete cycle was employed for extract preparation.

The antiviral activity of aerial-part extracts from nine species and leaf extracts from two species of the genus *Spiraea* was assessed here against the influenza A virus. Besides, the toxicity of dry extracts was analyzed, and the dry extracts that were most selective were identified (Table 1).

The assayed samples showed high (CC_50_ > 300 µg/mL) and moderate (CC_50_ > 158 µg/mL) cytotoxicity. The samples identified as the most toxic were *S. media* M1 and M2, *S. hypericifolia* H3 and H4, *S. trilobata* T6, *S. chamaedryfolia* L. Ch7 and Ch8, and *S. betulifolia* Pall. B11, *S. flexuosa* Fisch. ex Cambess. F12, and *S. ussuriensis* Pojark. U13. The least toxic sample, *S. salicifolia* S15, had a CC_50_ of 57.6 µg/mL.

All the dry *Spiraea* extracts showed an antiviral effect on influenza virus A. The half-maximal inhibitory concentration that halves viral activity (IC_50_) ranged from 5.9 to >300 μg/mL. The IC_50_ values indicated that the extracts from *S. salicifolia* S15 (5.9 μg/mL), *S. salicifolia* S14 (20.5 μg/mL), *S. crenata* L. (17 μg/mL), and *S. trilobata* (21 μg/mL) have the highest antiviral activity with the lowest IC_50_ values. The extracts of *S. chamaedryfolia* Ch 7, *S. flexuosa* F12, and *S. ussuriensis* U13 showed the least antiviral activity with the highest IC_50_ values (>300 μg/mL).

Judging by the SI, *Spiraea* samples were ranked as follows: Ch7 = F12 = U13 < T6 = Ch8 < M1 = M2 = H3 = C10 = B11 < H4 < T5 < C9 < S14 < S15. High selectivity was documented for the extracts of *S. salicifolia, S. crenata*, and *S. trilobata* (SI = 5–10). The highest selectivity was manifested by leaf (SI = 10) and aerial-part (SI = 8) extracts of *S. salicifolia* S14 and S15 and by the *S. crenata* C9 extract (SI = 6). The selectivity of *Spiraea* extracts proved to be equal to or greater than that of the antiviral agent rimantadine. The aerial-part extracts of *S. chamaedryfolia*, *S. flexuosa*, and *S. ussuriensis* (SI = 1 or 2) had low selectivity.

This study suggests that the extraction method plays a critical role in the extraction of valuable compounds. Extract preparation by repercolation revealed promising properties of the aerial part and leaf extracts of *S. salicifolia*. The antiviral activity exceeding the efficacy of rimantadine is reported for the first time for plants of the genus *Spiraea*. On the other hand, an *S. salicifolia* extract prepared by hot maceration has not shown an antiviral effect [34]. This method has probably not allowed us to isolate the desired class of compounds in the amounts needed. It should be noted that the raw material utilized to prepare this extract is similar to that used for the extracts employed in this study. On the contrary, extracts of *S. media* and *S. hypericifolia* prepared by maceration have higher antiviral activity than those prepared by repercolation [33,34]. Each plant species requires specific conditions for extraction of biologically active compounds in order to achieve optimal manifestation of pharmacological potential.

Moreover, the virus tested in our work is resistant to rimantadine, a well-known inhibitor of viral proton pump M2. Therefore, the target(s) and mechanism(s) of action of our extracts are distinct from M2 inhibition, and these extracts can serve as sources for the development of novel antivirals or their combinations.

### 3.2. Levels of Biologically Active Compounds Within Extract S15, Which Showed the Highest Antiviral Activity

Plants of the genus *Spiraea* are promising as a possible source of active substances with anti-influenza properties. Aerial parts of *Spiraea* species contain pharmacologically effective doses of biologically active compounds, such as flavonols, flavans, phenolic carboxylic acids, and tannins [18,21,35]. These substances possess a pronounced antiviral activity against influenza viruses [36]. The most commonly reported effects of polyphenols on viruses are inhibition of the viral replication cycle, suppression of viral genes, changed structure of virions, and blocked activity of RNA-dependent RNA polymerase (RdRp). These actions depend on the origin of a virus, on polyphenolic compounds, and on their interactions [37]. A synergistic interaction of ≥2 polyphenols can enhance the suppressive effect, even when they individually have hardly any antiviral activity [38,39].

Phytochemical screening of biologically active compounds within one of our extracts, which had the highest antiviral activity, indicates that the level of phenolic compounds in *S. salicifolia* S15 leaves reaches 147.95 mg/g. Of the phenolic compounds in the 40%-ethanolic extract prepared by repercolation, the predominant phenolic compounds were tannins (370.69 mg/g) and phenolcarboxylic acids (120.73 mg/g), whereas the concentrations of flavonoids (99.16 mg/g) and catechins (16.11 mg/g) were lower. In the *S. salicifolia* extracts prepared by hot maceration in a water bath [34], the total level of phenolcarboxylic acids, flavonols, and catechins is at a similar level or higher as compared to our extracts, whereas the total level of tannins exceeds that in our extracts prepared by repercolation.

**Figure 1:**
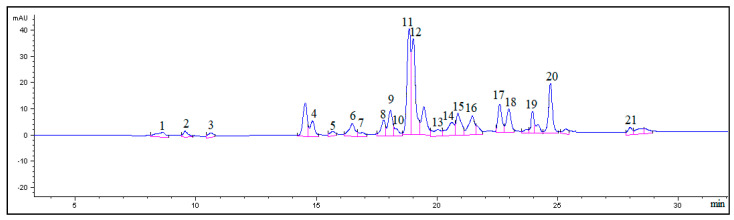
A chromatogram of the 40%-ethanolic extract of *S. salicifolia* S15 leaves at 340 nm. The *X*-axis shows retention time (min), whereas the *Y*-axis represents the detector signal in units of optical density. The peak numbers in the figure correspond to the peak numbers in Table 2.

Our analysis of the profile of phenolic compounds suggests that water-ethanol extracts of *S. salicifolia* S15 leaves contain at least 29 compounds (Figure 1). Of these, 21 compounds were identified based on UV spectral data and a comparison of retention time values of substance peaks between the chromatograms of the assayed samples and standard samples: five acids (chlorogenic, gentisic, caffeic, ferulic, and cinnamic) and 16 flavonoids quercetin and its glycosides (quercetin-3-glucuronoside, hyperoside, isoquercitrin, rutin, spiraeoside, avicularin, and quercitrin), kaempferol and its glycosides (nicotiflorin and astragalin), an isorhamnetin glycoside (isorhamnetin-3-rutinoside), luteolin glycosides (orientin and luteolin-7-glucoside), an apigenin glycoside (vitexin), and coumarin] (Table 2). We identified many of these compounds in our previous studies using liquid chromatography combined with high-resolution mass spectrometry [40]. In contrast to the extracts prepared by maceration [34], the extract of *S. salicifolia* S15 prepared by repercolation contained gentisic, ferulic, caffeic, and cinnamic acids, coumarin, luteolin-7-glucoside, quercetin-3-glucuronoside, quercitrin, and nicotiflorin, whereas *p*-coumaric acid was not detectable.

Major biologically active compounds in the *S. salicifolia* S15 leaf extract are flavonols: rutin (19.3 mg/g), isoquercitrin (16.6 mg/g), isorhamnetin-3-rutinoside (10.6 mg/g), and astragalin (9.5 mg/g). By contrast, major biologically active compounds in the *S. salicifolia* extract prepared by maceration include flavonols (isoquercitrin, astragalin, spiraeoside, quercetin, and kaempferol) [34]. It should be pointed out that the concentrations of the identified flavonoids and phenolcarboxylic acids, except for kaempferol, hyperoside, and spiraeoside, are higher in the S15 extract than in the extracts prepared by maceration [34].

Plant-derived compounds of the polyphenol class have been previously shown to possess antiviral activity. For instance, isoquercitrin, myricetin, and myricitrin from *Salicornia* spp. have manifested high efficacy in inhibiting HIV-1 infection and influenza virus neuraminidase [41]. Polyphenols from evergreen shrub *Maesa perlarius*, three proanthocyanidins [procyanidin B2, procyanidin C1, and epicatechin-(4β→8)-epicatechin-(4β→8)-epicatechin-(4β→8)-epicatechin], and two flavan-3-ols [(+)-catechin and (−)-epicatechin] have shown high anti-Ebola virus activities [42]. Furthermore, numerous flavonoids have been found to have therapeutic potential against COVID-19 owing to their targeting of the entry and replication of SARS-CoV-2 [43]. Flavonoids and other secondary metabolites of wild watermelon inhibit both early and late stages of influenza virus replication, whereas flavonols and flavanones can inhibit virus entry and have exerted replication-inhibitory effects on oseltamivir-resistant strains [44]. Therefore, our results agree with existing data suggesting that plant extracts containing flavonoids can inhibit influenza virus replication in cells.

In our article, we identified the major constituents of *Spiraea* extracts. These constituents may play a leading role in the virus-inhibitory properties of the whole extracts, or conversely, the minor constituent(s) may have the highest activity but are present at a low concentration within the extract and, therefore, could not give the extract a high enough activity. Further experiments are, therefore, needed to decipher their specific target(s) and mechanism(s) of action and to identify specific compounds responsible for this activity.

## 4. Conclusions

All the *Spiraea* extracts exerted an antiviral effect against the influenza A (H1N1) virus. The highest selectivity was found in leaf (SI = 10) and aerial-part (SI = 8) extracts of *S. salicifolia* S15 and in the *S. crenata* C9 extract (SI = 6). The observed selectivity of the *Spiraea* extracts is equal to or greater than the selectivity of the antiviral agent rimantadine. Aerial-part extracts of *S. chamaedryfolia, S. flexuosa*, and *S. ussuriensis* (SI = 1 or 2) showed low selectivity. Judging by IC_50_ values, the *S. salicifolia* S15 leaf extract has the strongest antiviral effect (IC_50_ = 5.9 μg/mL) and has the lowest toxicity (CC_50_ = 57.6 μg/mL) among the assayed plant tissue samples. Major biologically active compounds of the *S. salicifolia* S15 leaf extract include flavonol glycosides of quercetin (rutin and isoquercitrin), an isorhamnetin glycoside (isorhamnetin-3-rutinoside), and a kaempferol glycoside (astragalin). The selection of the best approaches to the extraction of biologically active compounds from plant raw materials is an important step in the search for promising sources of raw materials.

## Figures and Tables

**Table 1:** Antiviral activity of 40%-ethanolic extracts from *Spiraea* species, as prepared by the repercolation method.

ID	Species	Sampling Site	Plant Part (Ratio *)	Antiviral Activity		
				CC_50_, µg/mL	IC_50_, µg/mL	SI
M1	*S. media*	Experimental field of CSBG SB RAS	Aerial part (1:1:1)	>300	110	3
M2	*S. media*	Experimental field of CSBG SB RAS	Leaves	>300	87.2	3
H3	*S. hypericifolia*	Novosibirsk Oblast, Gorny settl. env.	Aerial part (whole branches with leaves and flowers)	>300	116	3
H4	*S. hypericifolia*	Experimental field of CSBG SB RAS	Aerial part (whole branches with leaves and flowers)	>300	73.8	4
T5	*S. trilobata*	Altai Territory, Medvedevka settl. env.	Aerial part (1:1.5: 1.5)	112	21	5
T6	*S. trilobata*	Experimental field of CSBG SB RAS	Aerial part (1:1:1)	>300	170	2
Ch7	*S. chamaedryfolia*	Rep. of Altai, Rep. of Altai, Amur settl. env.	Aerial part 1:1.3: 1.3	>300	>300	1
Ch8	*S. chamaedryfolia*	Experimental field of CSBG SB RAS	Aerial part (1:1:1)	>300	126	2
C9	*S. crenata*	Rep. of Altai, Ust-Koksa settl. env.	Aerial part (1:1.5:1.5)	98	17	6
C10	*S. crenata*	Novosibirsk Oblast, Shibkovo settl. env.	Aerial part (1:1:1)	86.2	>33	3
B11	*S. betulifolia*	Experimental field of CSBG SB RAS	Aerial part (1:1:1)	>300	110	3
F12	*S. flexuosa*	Experimental field of CSBG SB RAS	Aerial part (1:1.8: 1.8)	>300	>300	1
U13	*S. ussuriensis*	Amur Oblast, Zeya c. env.	Aerial part (1:1:1)	>300	>301	1
S14	*S. salicifolia*	Amur Oblast, Sergeevka settl. env.	Aerial part (1:1:1)	158	20.5	8
S15	*S. salicifolia*	Amur Oblast, Sergeevka settl. env.	Leaves	57.6	5.9	10
Oseltamivir carboxylate **				>100	0.3 ± 0.0	>333
Rimantadine **				286 ± 19	52 ± 6	6

* Annual branches of the plants were employed for the extraction: flowers, leaves, and stems in the ratio shown; IC_50_: half-maximal inhibitory concentration, i.e., the concentration that diminishes viral cytolytic activity by 50% relative to the control; CC_50_: half-maximal cytotoxic concentration, i.e., the concentration that kills 50% of cells in culture; env.: environs; c.: city; settl.: settlement; SI: the selectivity index, that is, the ratio of CC_50_ to IC_50_. ** IC_50_ and CC_50_ for the reference compounds oseltamivir carboxylate and rimantadine are presented in μM.

**Table 2:** The profile and levels of phenolic compounds in the *S. salicifolia* S15 leaf extract.

Peak No.	Compound	Retention Time (t_R_), min	Spectral Data λ_max_, nm	Level of Phenolic Compounds, mg/g
1	chlorogenic acid	8.5	244, 300 sh., 330	3.22
2	gentisic acid	9.6	240, 330	1.19
3	caffeic acid	10.6	240, 298 sh., 325	0.92
4	orientin	14.8	255, 270, 295 sh., 350	2.29
5	ferulic acid	15.4	235,295 sh., 320	0.80
6	vitexin	16.4	270, 340	4.63
7	coumarin	17.0	280, 315	1.36
8	luteolin-7-glucoside	17.7	250, 265 sh., 290 sh., 350	2.77
9	quercetin-3-glucuronoside	18.0	255, 260 sh., 290 sh., 355	5.39
10	hyperoside	18.3	255, 268 sh., 355	1.14
11	isoquercitrin	18.7	259, 266 sh., 358	16.60
12	rutin	19.0	255, 355	19.30
13	spiraeoside	20.3	255, 366	1.67
14	avicularin	20.8	260, 270 sh, 360	5.23
15	quercitrin	21.1	260, 330	6.25
16	astragalin	21.8	265, 300 sh., 350	9.46
17	nicotiflorin	22.4	260, 290 sh., 350	6.79
18	isorhamnetin-3-rutinoside	22.8	250, 265 sh., 280, 350	10.63
19	cinnamic acid	23.7	275	4.50
20	quercetin	24.7	255, 370	6.49
21	kaempferol	28.0	265, 365	0.75

sh.: shoulder; the peak numbers in the table correspond to the peak numbers in Figure 1.

## Data Availability

The original contributions presented in the study are included in the article, further inquiries can be directed to the corresponding author.
